# Efficacy of *Labisia pumila* and *Eurycoma longifolia* standardised extracts on hot flushes, quality of life, hormone and lipid profile of peri-menopausal and menopausal women: a randomised, placebo-controlled study

**DOI:** 10.29219/fnr.v64.3665

**Published:** 2020-09-03

**Authors:** Sasikala M. Chinnappan, Annie George, Malkanthi Evans, Joseph Anthony

**Affiliations:** 1 Biotropics Malaysia Berhad, Section U1Hicom Glenmarie, Industrial Park Shah Alam, Selangor, Malaysia; 2 KGK Science, London, ON, Canada

**Keywords:** Labisia pumila, Eurycoma longiolia, menopause, hot flush, hormonal imbalance

## Abstract

**Background:**

Interest in herbal medicines and non-hormonal therapies for the treatment of menopausal symptoms has increased since the publication of adverse effects of estrogen replacement therapy. Vasomotor symptoms are the most characteristic and notable symptoms of menopause.

**Objective:**

To investigate the changes in the frequency and severity of hot flush and associated vasomotor symptoms experienced by peri-menopausal and menopausal women supplemented with the herbal formulation (Nu-femme™) comprising *Labisia pumila* (SLP+^®^) and *Eurycoma longifolia* (Physta^®^) or placebo.

**Design:**

Randomised, double-blind, placebo-controlled, 24-week study enrolled 119 healthy women aged 41–55 years experiencing peri-menopausal or menopausal symptoms and supplemented with Nu-femme™ or placebo. The primary endpoint was comparative changes between treatment groups in the change in the frequency and severity of hot flushes. The secondary objectives were to assess the changes in the frequency and severity of joint pain, Menopause Rating Scale (MRS) and Menopause-Specific Quality of Life (MENQOL) questionnaire domain scores. Concentrations of serum hormone, lipid profile, bone markers, sleep quality and vitality were also studied as secondary objectives.

**Results:**

At week 12, significant (*P* < 0.01) improvements in hot flush symptoms were observed in Nu-femme™ and placebo groups. Even though there was no significant difference between groups, higher percentage of improvement, 65%, was seen in Nu-femme™ compared to 60% in placebo. Significant improvements (*P* < 0.001) in MRS and MENQOL scores at weeks 12 and 24 were observed in both groups, respectively. Luteinising hormone and follicle-stimulating hormone levels were significantly reduced (*P* < 0.05) at weeks 12 and 24, respectively, compared to baseline in the Nu-femme™ group, with no significant changes observed in the placebo group. There were significant (*P* < 0.05) reductions in serum low-density lipid and triglycerides levels at week 12 in Nu-femme™ group, but no changes seen in placebo group. At the end of week 24, changes in haematology and clinical chemistry parameters remained within normal clinical ranges in both groups.

**Conclusion:**

Herbal formulation consists of *L. pumila* and *E. longifolia* (Nu-femme™) may support reduction in hot flushes and improvements in hormone and lipid profile in healthy peri-menopausal and menopausal women.

## Popular scientific summary

- Combination of herbal extracts *L. pumila* (SLP+^®^) and *Eurycoma longifolia* (Physta^®^) found to be safe and well tolerated in perimenopuse and postmenopause population
- Use of herbal extracts *L. pumila* (SLP+^®^) and *Eurycoma longifolia* (Physta^®^) may support reduction in hot flushes, improvements in hormone balance and lipid profile in peri-menopausal and menopausal women

Menopause is the phase of a women’s life, in which there is a permanent cessation of menses and the end of reproductive potential, on average most women reach menopause between the ages of 45 and 55 years (1). Physiological changes, such as decreased production of ovarian hormones, indicate the transition from a reproductive to a non-reproductive state (2). Symptoms of menopause include hot flushes, night sweats, insomnia, depression, memory impairments, nervousness, bone and joint complaints and reduction in muscle mass. Women may experience these symptoms for several years prior to the menopause (3), with nearly 85% of postmenopausal women experiencing at least one menopause-related symptom in their lifetime (4, 5). Among these symptoms, hot flushes and night sweats, collectively referred as vasomotor symptoms, are commonly reported approximately 40–75% among women experiencing menopausal symptoms (6–8).

Effective symptom management is critical in the maintenance of quality of life (QoL) (9) and vitality, among menopausal women during this transition phase. Currently available options include hormone replacement therapies (HRTs), which provide treatment for postmenopausal–vasomotor symptoms, vaginal atrophy symptoms and sleep disturbances resulting from decreased oestrogen levels (10, 11). Recently, Brisdelle^®^, a selective serotonin reuptake inhibitor, was approved by the US Food and Drug administration and is the first non-hormonal treatment for menopausal hot flushes (9). While these therapies are effective in symptom reduction, they may be associated with undesirable side effects, such as headache, fatigue, nausea/vomiting, vaginal bleeding, breast pain and an increased risk of breast cancer (9, 10). Consequently, due to the risks associated with these treatments, a need remains for safe alternative therapies.

There has been a growing research interest in plant-based ingredients to mitigate menopause symptoms. A recent review of herbal products including black cohosh (*Cimicifuga racemosa*), wild yam (Diascorea), Dong quai (*Angelica sinensis*) and maca (Lepidium meyenii) conclude that larger, high-quality studies examining the safety and efficacy of these products are needed (12). Further, a systematic review and meta-analysis examined 62 randomised clinical trials reported the efficacy of plant-based therapies on menopause symptoms and found that specific phytoestrogen and herbal supplements were associated with reductions in hot flushes (13). However, due to the variation in study quality and heterogeneity of results, high-quality studies examining the role of plant-based supplements on menopausal symptoms are needed.

The investigational product used in this study, Nu-femme™, is a herbal supplement composed primarily of standardised water extracts of *Labisia pumila* (SLP+^®^) and *Eurycoma longifolia*(Physta^®^), locally known as ‘Kacip Fatimah’ and ‘Tongkat Ali’, respectively. These herbs have been commonly used for centuries as traditional medicines for reproductive health, promoting energy and enhancing mental alertness (14–18). The effectiveness of *L. pumila* is attributed to the flavonoid and phenolic contents; as well phytoestrogenic properties (19), *L. pumila* has been used traditionally to ease childbirth, postpartum rejuvenation (20) and relieve from menopausal symptoms (21). *E. longifolia*, known as Malaysian ginseng, contains a variety of metabolites including eurycomanone and a 4.3 kDa peptide, which influence testosterone production. *E. longifolia* has been used by men to enhance health and libido (22), and the root of *E. longifolia* is often taken as a health tonic after childbirth (23). A recent study has concluded that *E. longifolia*could be beneficial in managing hormonal imbalance in ovariectomised rats (24). Both herbs, *L. pumila* and *E. longifolia*, had shown some benefits in managing hormonal imbalance and symptoms associated with menopause, but there is no study to investigate the effectiveness of the combination of these two herbs in menopause population. Combination of *L. pumila* and *E. longifolia* may able to modulate estrogenic and androgenic effect and could be beneficial in menopausal symptoms management. The objectives of this randomised, double-blind, placebo-controlled, two-arm parallel study were to investigate the changes in the frequency and severity of vasomotor symptoms (hot flushes) experienced by peri-menopausal and menopausal women supplemented with Nu-femme™ or placebo. Other outcomes assessed included joint pain symptoms, hormonal and lipid profile, bone markers of health, sleep quality and vitality, and safety of Nu-femme™ over the 24-week study period.

## Materials and methods

This study was conducted in accordance with the Guidelines for Good Clinical Practice (ICH-6) and the Declaration of Helsinki. Institutional Review Board (IRB) approval was obtained from IRB Services, Aurora, ON, Canada on 15 September 2014 prior to initiation of any study-related activities. The IRB reviewed the protocol, medical ethics, informed consent, advertisement, reimbursement and compliance to protocol. This study was conducted at KGK Sciences Inc., London, Ontario, Canada. A written informed consent was obtained from volunteers prior to all study procedures. The recruitment and follow-up took place from 17 December 2014 to 27 September 2018.

### Study design

This was a randomised, double-blind, placebo-controlled, two-arm parallel study with a 24-week safety and efficacy period. The allocation ratio of participants in each of the comparison groups was 1:1. Participants were required to make a total of five visits to the clinical trial sites chosen for this study in Montreal, Quebec, Orlando, Toronto and at KGK Sciences Inc., London, ON, Canada.

At screening (visit 1), eligibility was assessed for peri-menopausal and menopausal women participants. Screening assessments included medical history review, vital signs, anthropometric measures, haematology and clinical chemistry parameters. Blood was also sampled for thyroid stimulating hormone (TSH) and prolactin levels. Eligible participants returned for their baseline visit (visit 2, day 0) and were randomised to one of the two arms of the study. Baseline assessments included vital signs, anthropometric measures, urine pregnancy, physical exam tests, lipid profile, estradiol 17β, sex hormone binding globulin (SHBG), luteinising hormone (LH), follicle-stimulating hormone (FSH), total testosterone, free testosterone, bone marker, that is, amino-terminal cross linked telopeptide of type 1 collagen (NTX) and bone-specific alkaline phosphatase (BSAP) analysis. Additionally,administration of vitality index, Menopause-Specific Quality of Life (MENQOL) and Menopause Rating Scale (MRS) questionnaires and the review of the subject symptom diary were performed at visit 1, as well as every subsequent visit. Ultrasound and Pap smear tests were performed between screening and baseline visits. At visit 3 (week 6, day 43), assessments included anthropometrics measures, vital signs, analysis of lipid panel and androgenic side effects. Adverse events (AEs) were reviewed at every subsequent visit. At visit 4 (week 12, day 85), participants returned for vital signs, anthropometric measures and assessments of androgenic side effects. In addition, blood was sampled for lipid profile, estradiol 17β, SHBG, LH, FSH, total testosterone and free testosterone assessments. On visit 5 (week 24, day 169), participants had their vital signs, and anthropometric measures taken, ultrasound test, androgenic side effects, clinical chemistry parameters and haematology assessed. Blood was also sampled for lipid profile, estradiol 17β, SHBG, LH, FSH, total testosterone, free testosterone and bone marker (NTX and BSAP) analysis.

### Participants

**Fig. 1:**
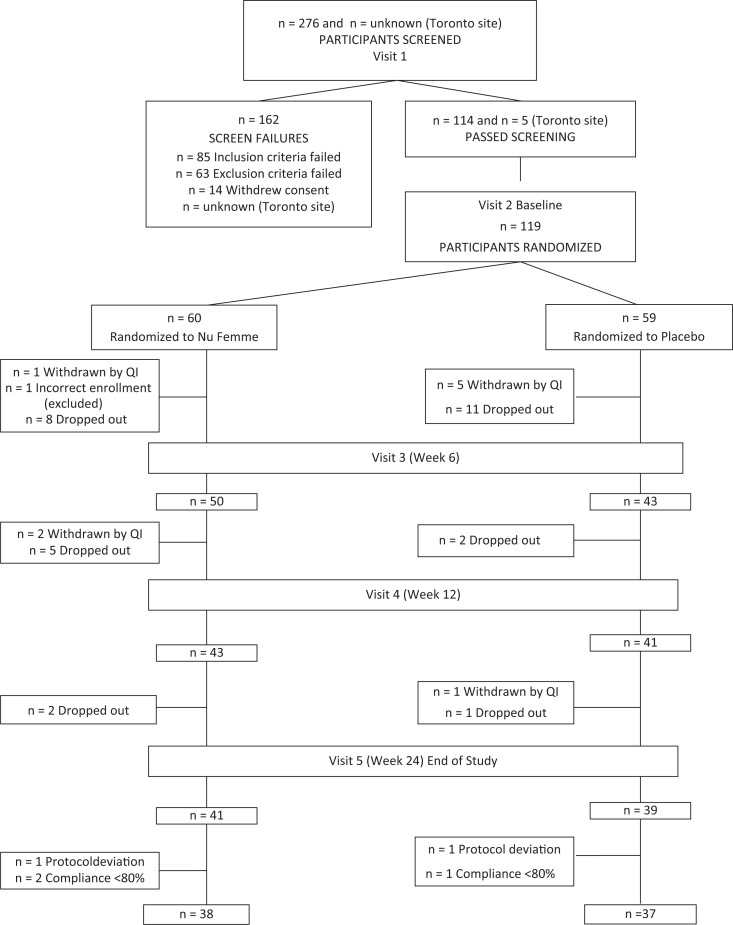
A total of 276 participants were screened and 119 participants were enrolled in this study, randomised to either Nu-femme™ or placebo groups. Of the 75 participants included in the analysis, 38 were in the Nu-femme™ group and 37 were in the placebo group.

Study participants were recruited from the general population by online advertising, recruiting and available clinical trial databases. The inclusion and exclusion criteria are described in Table 1. A total of 276 participants were screened and 119 eligible participants were enrolled in this study, with 60 participants in the Nu-femme™ and 59 in the placebo groups (Fig. 1).

**Table 1:** The inclusion and exclusion criteria

Inclusion criteria	Exclusion criteria
Females between 40 and 55 years of age	Positive mammogram or history/diagnosis of breast cancer
Peri-menopause (irregular menstrual cycles > 3 months) or cessation of menstrual period for at least 3 months within the last 12 months or women in menopause (cessation of menstrual period for at least 12 months)	History of uncontrolled hypertension or hyperlipidemia History or diagnosed with cardiovascular, hepatic, renal, gastrointestinal, pulmonary or endocrine system diseases
Peri-menopausal women with an endometrial stripe <8 mm at screening	Uncontrolled diabetes (type I or II)
Menopausal women with an endometrial stripe <5 mm at screening	Uncontrolled and/or untreated thyroid disorder
Not required for subjects without an intact uterus	Diagnosed autoimmune condition(s), immunodeficiency or gynaecological diseases
Women with an intact cervix with a normal Pap smear within 12 months of screening	Clinically significant mental depression
Experienced menopausal transition symptoms (e.g. hot flushes, sweating, sleep disturbance, migraine, anxiety, vaginal dryness and sexual problems)	Major surgery within the year prior to randomisation, except for cholecystectomy and appendectomy
Minimum of four hot flushes per day or 28 per week	
MRS total scores ≥ 17, indicating moderate or severe menopausal symptoms	Smoked more than 15 cigarettes per day
TSH screening performed	History of alcohol or drug abuse Abnormal vaginal bleeding within the previous 2 years prior to randomisation Uterine fibroids more than 2 cm or endometriosis Pregnant with positive urine pregnancy test results

### Investigational product

The investigational product comprised 200 mg *L. pumila* (SLP+^®^), 50 mg *E. longifolia* (Physta^®^) and 100 mg maltodextrin as the excipient (non-medicinal ingredient). The placebo contained maltodextrin (300 mg). The *L. pumila* extract used in investigational product is commercially available from Biotropics Malaysia under the trade name of SLP+^®^. It is a water extract of the leaves and stems of *L. pumila*, standardised based on the specification of not less than 0.1% gallic acid. The *E. longifolia* extract used in investigational product is also commercially available from Biotropics Malaysia under the trade name of Physta^®^. It is a water extract of the roots of *E. longifolia*, standardised based on the specification of 0.8-1.5% eurycomanone, not less than 22% of total protein, not less than 30.0% of total polysaccharide and not less than 40.0% of glycosaponin. Both investigational product and placebo were manufactured under good manufacturing practice in the facility of Biotropics Malaysia Berhad. Participants were instructed to consume two capsules daily in the morning after breakfast. The daily total intake of *L. pumila* (SLP+^®^) and *E. longifolia* (Physta^®^) is 400 and 100 mg, respectively. These doses were selected as these were used in past clinical trials and known to be safe (16, 25).

### Outcome measures

The primary and secondary outcomes measured were assessed by questionnaires applied at visits 1–5. The primary outcome measure for this study was changes in the hot flush symptom score based on the frequency and severity of hot flushes from baseline (pre-treatment) to week 12 between treatment groups. The hot flush symptom score was calculated by assigning a number to the intensity of the hot flushes (1, mild; 2, moderate; 3, severe; 4, very severe), and multiplying this by the daily frequency of the hot flush of that intensity.

The secondary objectives were to assess changes in the joint pain symptom score based on the frequency and severity of joint pain from baseline (pre-treatment) to week 12. The joint pain symptom score was calculated in the same fashion as the hot flush symptom score. Other outcome measures were changes in the hot flush symptom score from baseline (pre-treatment) to week 24 and from week 12 to week 24, changes in the joint pain symptom score from baseline (pre-treatment) to week 24 and from week 12 to week 24, MRS and MENQOL domain scores, individual scores as well as total scores, changes from baseline (pre-treatment) to 6, 12 and 24 weeks and differences between groups at weeks 6, 12 and 24. Changes in serum hormone (estradiol-17b, FSH, LH, total testosterone, free testosterone and bioavailable testosterone), serum lipid profile (total cholesterol, high density lipid (HDL), low density lipid (LDL) and triglycerides) concentrations, sleep problems (assessed by the sum of the scores of question #3 of the MRS and question #14 of the MENQOL) and vitality from baseline (pre-treatment) to week 12 and from baseline (pre-treatment) to week 24 and from week 12 to week 24, using the vitality index are other outcome measured in the study.

Safety and tolerability of the investigation product were assessed through changes in complete blood count (CBC), comprehensive metabolic panel (CMP), pelvic ultrasound at baseline and end of study visit, and vital signs measured at all visits.

### Compliance

The dispensed study product compliance diaries were returned to the clinic, and all participants who were non-compliant with their diaries were reminded of their obligations regarding the appropriate study compliance. Compliance was assessed by counting the returned unused test product at each visit calculated by determining the number of dosage units taken divided by the number of dosages expected to have been taken, multiplied by 100.

### Sample size

Sample size calculations were based on comparing the primary endpoint (12-week change in hot flush score) between Nu-femme™ and placebo groups with an unpaired Student *t*-test. A within-group standard deviation (SD) estimate of 7.1 for hot flush score values was obtained from a previous study using this score as the primary endpoint (26). To have 80% power to obtain *P* < 0.05 when comparing score changes between Nu-femme™ and placebo for a true effect size of 4.5 score unit difference, the within-group SD was 7.1, resulting in 40 subjects per group. A 20% attrition rate was considered relevant, thus resulting in 50 subjects per group.

### Randomisation

Participants were identified by their initials and their date of birth and were assigned a participant number at their screening visit (visit 1). If the potential participant met all the inclusion criteria and did not meet any of the exclusion criteria at baseline (visit 2), a randomisation number was assigned and the participant was randomised to one of the two arms of the study by a blinded investigator per the order of the randomisation list generated by www.randomization.com. Investigators, other site personnel and participants were blinded to the product. The investigational product was stored correctly, and an accurate record of its dispensing to the study participant was maintained.

### Statistical analysis

The per-protocol analysis included subjects who consumed at least 80% of either product, did not have any major protocol deviation and completed all study visits and procedures connected with the measurement of the primary endpoints. Subgroup analysis was performed for the primary outcome based on hot flush score >20 and high levels of free testosterone at baseline, that is, more than 6.9 pmol/L.

Dependent variables were tested for normality and skewed variables were log-transformed before analysis. Intractably non-normal variables were analysed using appropriate non-parametric methods. Possible differences at baseline and week 12 between the two groups were assessed by independent two-sample *t*-tests, and Welch–Satterthwaite’s correction was performed when variances of the two groups were significantly different at baseline.

Analysis of covariance (ANCOVA) was conducted to evaluate the change in hot flush scores from baseline to weeks 12 and 24. The ANCOVA model included the baseline value as a covariate with a group as the fixed effect while controlling for trial centre location. Four covariance structures were tested separately and the one that provided the smallest Akaike’s information criteria was selected. The covariance structures were compound symmetry (CS), heterogeneous CS, first-order autoregressive and heterogeneous autoregressive. For MRS and MENQOL, study week and group by study week interaction were also included as fixed effects. Treatment group and week number were treated as categorical variables (factors), while the dependent variable was treated as a continuous variable in the ANCOVA model. A linear contrast statement was constructed from the model to obtain the *P*-values for between groups and within-group comparisons. Least squares means (LS-Means) and standard errors of LS-Means (SEM) were obtained from the model.

Probabilities ≤0.05 were considered statistically significant. All statistical analyses were completed using the R Statistical Software Package Version 3.5.2 (R Core Team, 2018) for Microsoft Windows.

## Results

### Participant baseline characteristics

Over the period of the study, 39 participants were terminated early from the study. One participant was found to have been enrolled incorrectly after randomisation and was excluded from the study. Two participants had major protocol deviations (incomplete study diary and did not meet the inclusion criteria), and three participants had overall compliance less than 80% (assessed based on study product compliance diaries) and were excluded from the analysis. For analysis, 38 participants were included in the Nu-femme™ group and 37 participants in the placebo group (Fig. 1). The overall mean compliance for the Nu-femme™ group was 96.71 and 96.84% for the placebo group, which was not significantly different. Participants in the study ranged from 41 to 55 years of age and were predominantly Western or Eastern European White in the Nu-femme™ (76.7%) and placebo (71.2%) groups. There were no differences in the number of participants who smoked currently, did not smoke or were ex-smokers. There were no differences whether participants were peri-menopausal or menopausal (Table 2).

**Table 2:** Participant demographics comparing Nu-femme™ and placebo groups

	Nu-femme™ (*n* = 38)	Placebo (*n* = 37)	Between-group P*
**Age (years), mean ± SD**	51.53 ± 2.53	51.41 ± 2.74	0.970
**Weight (kg), mean ± SD**	74.69 ± 17.16	78.14 ± 19.42	0.494
**BMI (kg/m^2^), mean ± SD**	28.24 ± 6.22	29.53 ± 6.82	0.464
**Race, n**			
Black or African American	1	0	0.229
Central American	0	1	
East Asian	0	1	
Eastern European White	2	4	
Middle Eastern	1	0	
North American Indian	1	0	
South American	5	1	
Western European White	28	30	
Ethnicity, **n**			
Hispanic or Latino	5	2	0.430
Not Hispanic or Latino	33	35	
**Study site, n**			
London, ON	30	33	0.622
Montreal	1	1	
Orlando	4	3	
Quebec	1	0	
Toronto	2	0	
**Smoking status, n**			
Smoker	11	14	0.517
Non-smoker	40	34	
Ex-smoker	8	11	
**Menopause status, n**			
Menopausal	19	21	0.557
Peri-menopausal	19	16	

* For continuous parameters, between-group *P*-values generated from ANOVA models with group as a fixed effect. For categorical parameters, between-group *P*-values generated by Chi-square or Fisher’s exact (two tail) tests as appropriate.

SD, standard deviation.

There were no significant between-group differences observed in vitals and anthropometric measurements at baseline. At screening, there were no significant between-group differences in haematological parameters. All clinical chemistry parameters values, concentrations of hormones, prolactin and TSH were within the normal clinical ranges. All participants were healthy as assessed by anthropometric, vital signs, CBC and CMP measurements.

### Primary endpoint

**Fig. 2:**
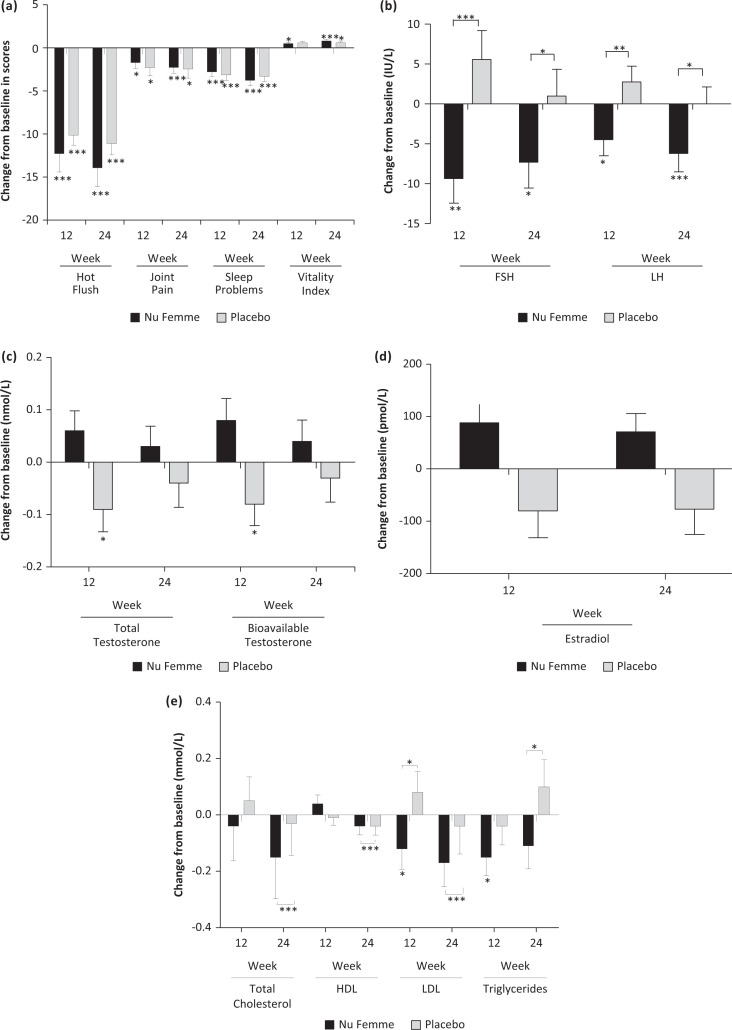
(A) The Nu-femme™ and placebo groups showed significant within-group differences in reduction of hot flushes, joint pain, sleep problem and increase in vitality index. No significance between-group difference was observed (***P < 0.001; *P < 0.05). (B) The Nu-femme™ group showed a significant decrease in follicle-stimulating hormone (FSH) and luteinising hormone (LH) from baseline to weeks 12 and 24. There was a significant between-group difference in the changes of FSH and LH levels at weeks 12 and 24 (***P < 0.001; **P < 0.01; *P < 0.05). (C) The placebo group showed a significant decrease in total and bioavailable testosterone from baseline to week 12. Despite a lack of significance, the Nu-femme™ group showed an increasing trend for these hormones, but there was no significant between-group difference in the changes of total testosterone and bioavailable testosterone (*P < 0.05). (D) Despite a lack of significance, the Nu-femme™ group demonstrated an increasing trend in estradiol levels at weeks 12 and 24, in contrast to the decreasing trend demonstrated in placebo group from baseline to weeks 12 and 24. (E) The Nu-femme™ group demonstrated a significant reduction in serum LDL and triglycerides levels at week 12 compared to baseline. There was a significant between-group difference in the changes of total cholesterol, serum HDL, triglycerides at week 24 and serum LDL at week 12 and week 24 (***P < 0.001; *P < 0.05).

Participants supplemented with Nu-femme™ or placebo showed significant (*P* < 0.001) improvements of 65 and 60%, respectively, in hot flush symptoms after 12 weeks, with no significant between-group and comparative changes between groups at week 12 (Table 3 and Fig. 2A).

**Table 3:** Summary of findings from the quality of life questionnaires

	Before supplementationMean ± SD		P*	After supplementation – week 12 Mean ± SD (*n*)		P*	After supplementation – week 24 Mean ± SD (*n*)		P*
	Nu-femme™ (*n* = 38)	Placebo (*n* = 37)		Nu-femme™ (*n* = 38)	Placebo (n = 37)		Nu-femme™ (*n* = 38)	Placebo (*n* = 37)	
Hot flush symptoms	19.00 ± 11.83	16.81 ± 9.27	0.273	6.72 ± 4.92^***^	6.66 ± 6.16^***^	0.596	5.06 ± 4.51^***^	5.79 ± 5.63^***^	0.825
Joint pain symptoms	3.32 ± 4.63	5.25 ± 6.94	0.058	1.62 ± 2.75^**^	3.09 ± 5.28^**^	0.095	1.05 ± 2.00^***^	2.89 ± 6.09^**^	0.334
MRS score	23.87 ± 6.53	25.41 ± 8.49	0.648	14.34 ± 7.12^***^	13.97 ± 10.14^***^	0.451	12.26 ± 6.92^***^	12.32 ± 7.97^***^	0.890
MENQol score	134.13 ± 38.60	137.84 ± 38.35	0.678	96.03 ± 36.21^***^	97.62 ± 40.60^***^	0.907	89.03 ± 41.21^***^	86.73 ± 40.67^***^	0.807
Vitality score	3.88 ± 1.16	3.91 ± 1.05	0.537	4.39 ± 0.95^**^	4.46 ± 0.92	0.762	4.76 ± 0.91^***^	4.45 ± 1.07^*^	0.199

For visit comparisons, between-group *P*-values generated by independent *t*-test or Wilcoxon Rank Sum test (Mann–Whitney *U*). For *t*-tests, Satterthwaite’s correction was conducted when the variances of the two groups were significantly different. Between-group *P*-value for the change from baseline (day 0) was generated from ANCOVA with baseline as a covariate and group and site as fixed effects, or Wilcoxon Rank Sum test (Mann–Whitney *U*). Ties excluded from Wilcoxon tests.

*n*, number; SD, standard deviation.

^*^P < 0.05, ^**^P < 0.01, ^***^P < 0.001, significant within-group.

### Secondary outcomes

Participants supplemented with Nu-femme™ or placebo showed 73% (*P* < 0.001) and 66% (*P* < 0.001), respectively, significant improvements in hot flush symptoms after 24 weeks with no significant between-group differences at the end of the study. Furthermore, participants supplemented with Nu-femme™ or placebo showed 68% (*P* < 0.001) and 47% (*P* < 0.01), respectively, significant improvements in joint pain symptoms after 24 weeks with no significant between-group differences at the end of the study (Table 3 and Fig. 2A).

Thus, total scores for MRS and MENQOL assessed at all time points in the study showed significant improvements (*P* < 0.001) in both Nu-femme™ and placebo groups, without any significant between-group differences (Table 3). The Nu-femme™ group showed a significant within-group increase in vitality at week 12 (*P* < 0.01) and week 24 (*P* < 0.001), whereas a significant improvement (*P* < 0.05) in vitality scores was only observed at week 24 for the placebo group. Based on the scores obtained in the vitality index, Nu-femme™ showed increase of 21%, whereas the increase in the placebo was only 14% at end of week 24 (Table 3 and Fig. 2A).

There was no significant change in the hormonal profile between groups at weeks 12 and 24, but a significant reduction in serum FSH and LH levels in the Nu-femme™ group was observed. At weeks 12 (*P* < 0.01) and 24 (*P* < 0.05), serum FSH in the Nu-femme™ group significantly decreased compared to baseline. Similarly, serum LH was significantly dropped at week 12 (*P* < 0.05) and week 24 (*P* < 0.001) compared to baseline in Nu-femme™ group (Table 4). There was significant between-group differences in serum FSH level at week 12 (*P* < 0.001) and week 24 (*P* < 0.05), and LH level at week 12 (*P* < 0.01) and week 24 (*P* < 0.05) (Fig. 2B). Serum total testosterone (*P* < 0.05) and bioavailable testosterone (*P* < 0.05) levels showed a significant decrease in the placebo group from baseline to week 12 (Table 4). Despite the lack of significance, Nu-femme™ supplementation enhanced free testosterone concentrations by 40% over baseline values, compared to a reduction in the placebo group (Table 4 and Fig. 2C) at week 12. However, SHBG levels remained constant in both Nu-femme™ and placebo groups. Likewise, Nu-femme™ supplementation demonstrated an increasing trend in oestradiol levels compared to the reduction in the placebo group (Table 4 and Fig. 2D).

**Table 4:** Serum hormone, lipid profile and NTX concentration of participants supplemented with Nu-femme™ or placebo

	Before supplementation Mean ± SD		P*	After supplementation – week 12 Mean ± SD		P*	After supplementation – week 24 Mean ± SD		P*
	Nu-femme™ (*n* = 38)	Placebo (*n* = 37)		Nu-femme™ (*n* = 38)	Placebo (*n* = 37)		Nu-femme™ (*n* = 38)	Placebo (*n* = 37)	
FSH (IU/L)	71.51 ± 45.70	59.13 ± 36.06	0.270	60.30 ± 40.91^**^	64.71 ± 28.47	0.257	64.20 ± 40.54^*^	60.13 ± 29.35	0.996
LH (IU/L)	38.39 ± 22.39	30.73 ± 17.11	0.127	31.87 ±17.21^*^	33.50 ± 16.82	0.474	32.19 ± 15.61^***^	30.75 ± 15.14	0.816
Total testosterone (nmol/L)	0.47 ± 0.28	0.60 ± 0.37	0.106	0.54 ± 0.25	0.50 ± 0.31^*^	0.856	0.49 ± 0.26	0.56 ± 0.39	0.681
Bioavailable testosterone (nmol/L)	0.45 ± 0.30	0.59 ± 0.38	0.167	0.54 ± 0.25	0.50 ± 0.31^*^	0.856	0.49 ± 0.26	0.56 ± 0.39	0.522
Free testosterone (pmol/L)	6.27 ± 5.20	6.93 ± 4.48	0.360	8.73 ± 11.64	6.93 ± 3.70	0.732	6.19 ± 3.94	7.03 ± 4.25	0.960
Estradiol-17b (pmol/L)	70.92 ± 67.96	153.33 ±296.95	0.601	159.05 ± 233.48	73.09 ± 114.48	0.571	141.79 ± 225.27	76.63 ± 83.53	0.987
SHBG (nmol/L)	71.50 ± 38.70	74.89 ± 45.49	0.945	70.10 ± 60.30	71.65 ± 45.36	0.928	71.70 ± 37.04	74.61 ± 44.70	0.488
Total cholesterol (mmol/L)	5.43 ± 1.12	5.43 ± 0.70	0.812	5.38 ± 0.96	5.46 ± 0.51	0.266	5.27 ± 0.97	5.38 ±0.86	0.603
LDL cholesterol (mmol/L)	3.21 ± 0.92	3.25 ± 0.74	0.845	3.09 ± 0.91^*^	3.32 ± 0.63	0.114	3.04 ± 0.85	3.21 ± 0.81	0.386
HDL cholesterol (mmol/L)	1.79 ± 0.43	1.62 ± 0.48	0.043	1.81 ± 0.40	1.60 ± 0.42	0.042	1.74 ± 0.39	1.58 ± 0.45	0.091
Triglycerides (mmol/L)	1.18 ± 0.52	1.23 ± 0.54	0.882	1.04 ± 0.41^*^	1.18 ± 0.52	0.132	1.07 ± 0.51	1.32 ± 0.72	0.07
N-terminal telopeptide (nM BCE)	16.79 ± 4.72	16.39 ± 4.53	0.812	NT	NT	NT	16.14 ± 4.75	16.32 ± 4.78	0.921
BSAP (µ/L)	76.35 ± 21.30	74.78 ± 19.83	0.657	NT	NT	NT	78.03 ± 20.94	77.43 ± 19.74	0.482

For visit comparisons, between-group *P*-values generated by independent *t*-test or Wilcoxon Rank Sum test (Mann–Whitney *U*). For *t*-tests, Satterthwaite’s correction was conducted when the variances of the two groups were significantly different. Between-group *P*-value for the change from baseline (day 0) was generated from ANCOVA with baseline as a covariate and group and site as fixed effects, or Wilcoxon Rank Sum test (Mann–Whitney *U*). Ties excluded from Wilcoxon tests. BCE: bone collagen equivalents *n*, number; SD, standard deviation; NTX: amino-terminal cross linked telopeptide of type 1 collagen; FSH: follicle-stimulating hormone; LH: luteinising hormone; SHBG: sex hormone binding globulin; LDL: low-density lipid; HDL: high density lipid; BSAP: bone-specific alkaline phosphatase.

^*^P < 0.05, ^**^P< 0.01, ^***^P < 0.001, significant within-group; within-group *P*-values generated by the paired *t*-test or Wilcoxon Signed Rank test. Ties or zeros excluded from Wilcoxon tests.

Even though there was no significant difference between groups in lipid panel, supplementation of Nu-femme™ ameliorated trends in the lipid panel, with the reduction in total cholesterol at week 24 significantly higher (*P* < 0.001) compared to the placebo group. Similarly, there was a significant decrease in serum LDL levels at week 12 (*P* < 0.01) and week 24 (*P* < 0.001) and serum triglycerides at week 24 (*P* < 0.05) observed in the Nu-femme™ group compared to changes in the serum LDL and triglycerides levels in placebo group at these time points (Fig. 2E).

Total bone mineral density using markers of bone health, NTX and BSAP showed no significant between-group differences. However, levels of NTX decreased by 4.6% in the Nu-femme™ group compared to 0.5% in the placebo group (Table 4), whereas BSAP showed a significant 4.0% increase in the placebo group (*P* < 0.05).

### Subgroup analysis

**Fig. 3:**
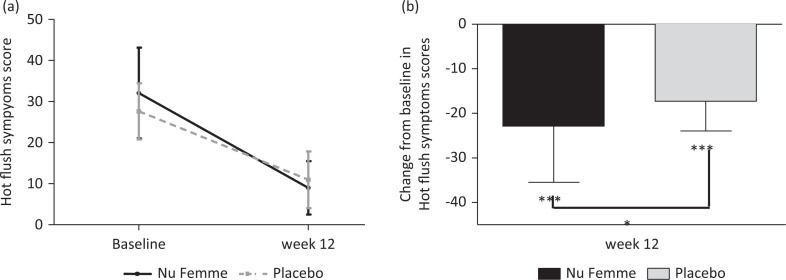
Both Nu-femme™ and placebo groups showed a significant improvement in hot flush symptoms, with Nu-femme™ supplementation alleviated hot flush symptoms by a significant 15% over the placebo in a subset of women who had a hot flush symptom score of >20 at baseline (***P < 0.001; *P < 0.05).

**Fig. 4:**
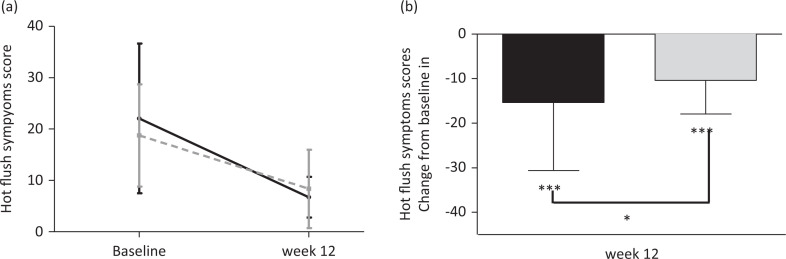
Both Nu-femme™ and placebo groups comprising participants with high levels of free testosterone at baseline showed a significant improvement in hot flush symptoms, with Nu-femme™ supplementation alleviated hot flush symptoms by a significant 15% over the placebo (***P < 0.001; *P < 0.05).

The analysis showed that while both Nu-femme™ and placebo groups showed a significant improvement in hot flush symptoms, Nu-femme™ supplementation alleviated hot flush symptoms by a significant 15% over the placebo (*P* < 0.05) in a subset of women (*n* = 19 in the Nu-femme™ and *n* = 21 in the placebo group) who had a hot flush symptom score of >20 at baseline (Fig. 3). Furthermore, both Nu-femme™ (*n* = 15) and placebo groups (*n* = 15) comprising participants with high levels of free testosterone at baseline showed a significant improvement in hot flush symptoms, with Nu-femme™ supplementation alleviating hot flush symptoms by a significant 15% over the placebo (*P* < 0.05) (Fig. 4).

### Safety parameters

At the end of week 24, changes observed in haematology and clinical chemistry parameters remained within clinically normal ranges in both groups (Table 5).

**Table 5:** CBC, CMP and safety parameters assessed in participants

Item	Reference value	Group	Screening (week 0)	Week 24
Haemoglobin levels (g/L)	110–147	Nu-femme™	133.07 ± 8.73	130.48 ± 11.01^*^
		Placebo	132.86 ± 8.60	131.49 ± 10.81
Haematocrit (L/L)	0.33–0.44	Nu-femme™	0.40 ± 0.02	0.39 ± 0.03^*^
		Placebo	0.40 ± 0.02	0.39 ± 0.03
White blood cell count (×10^9^/L)	3.2–9.4	Nu-femme™	5.96 ± 1.65	5.65 ± 1.70
		Placebo	5.90 ± 1.54	5.79 ± 1.60
Red blood cell count (×10^12^/L)	3.8–5.2	Nu-femme™	4.53 ± 0.29	4.46 ± 0.32
		Placebo	4.46 ± 0.32	4.45 ± 0.37
Mean corpuscular volume (fL)	76–98	Nu-femme™	88.56 ± 5.14	88.08 ± 5.43^*^
		Placebo	89.50 ± 4.45	88.53 ± 3.53
Mean corpuscular haemoglobin (pG)	24–33	Nu-femme™	29.46 ± 1.89	29.29 ± 2.24^*^
		Placebo	29.86 ± 1.67	29.56 ± 1.60
Mean corpuscular haemoglobin concentration (g/L)	313–344	Nu-femme™	332.72 ± 7.18	332.46 ± 8.95
		Placebo	333.71 ± 6.78	333.67 ± 8.20
Red blood cell distribution width (%)	12.5–17.3	Nu-femme™	13.84 ± 1.10	13.77 ± 1.09
		Placebo	13.79 ± 0.96	13.78 ± 0.66^*^
Platelets count (×10^9^/L)	155–372	Nu-femme™	282.72 ± 57.51	272.64 ± 61.88
		Placebo	274.83 ± 66.92	275.67 ± 82.30
Absolute neutrophil count (×10^9^/L)	1.4–6.3	Nu-femme™	3.47 ± 1.29	3.26 ± 1.25
		Placebo	3.22 ± 1.06	3.21 ± 1.13
Absolute lymphocyte count (×10^9^/L)	1.0–2.9	Nu-femme™	1.88 ± 0.49	1.79 ± 0.55
		Placebo	2.03 ± 0.61	1.95 ± 0.62
Absolute monocyte count (×10^9^/L)	0.2–0.6	Nu-femme™	0.45 ± 0.14	0.42 ± 0.15
		Placebo	0.46 ± 0.12	0.43 ± 0.14
Absolute eosinophil count (×10^9^/L)	0.0–0.5	Nu-femme™	0.15 ± 0.08	0.17 ± 0.12^*^
		Placebo	0.17 ± 0.11	0.16 ± 0.11
Absolute basophil count (×10^9^/L)	0.00–0.09	Nu-femme™	0.02 ± 0.04	0.02 ± 0.04
		Placebo	0.02 ± 0.04	0.02 ± 0.04
Fasting blood glucose (mmol/L)	3.6–6.0	Nu-femme™	5.08 ± 0.59	5.21 ± 0.91
		Placebo	5.22 ± 0.50	5.34 ± 0.59
Creatinine (mmol/L)	50–100	Nu-femme™	65.74 ± 11.93	66.08 ± 12.79
		Placebo	65.12 ± 11.64	66.03 ± 11.11
Estimated glomerular filtration rate (mL/min)	≥90	Nu-femme™	91.33 ± 13.52	90.54 ± 14.24
		Placebo	92.27 ± 14.08	91.13 ± 13.39
Urea (mmol/L)	2.5–8.1	Nu-femme™	4.80 ± 0.97	4.99 ± 1.06
		Placebo	4.65 ± 1.27	4.72 ± 1.38
Sodium (mmol/L)	136–146	Nu-femme™	142.43 ± 2.22	142.12 ± 2.09
		Placebo	142.27 ± 2.36	141.62 ± 2.91
Potassium (mmol/L)	3.4–5.0	Nu-femme™	4.57 ± 0.43	4.43 ± 0.32^*^
		Placebo	4.57 ± 0.44	4.36 ± 0.45^**^
Calcium (mmol/L)	2.15–2.60	Nu-femme™	2.39 ± 0.09	2.33 ± 0.07^**^
		Placebo	2.37 ± 0.09	2.36 ± 0.09
Chloride (mmol/L)	95–108	Nu-femme™	103.72 ± 3.16	103.36 ± 2.78
		Placebo	103.56 ± 2.91	102.47 ± 3.16^*^
Total bilirubin (µmol/L)	<23	Nu-femme™	8.43 ± 4.58	7.65 ± 3.48^**^
		Placebo	7.88 ± 4.23	7.34 ± 3.56
Albumin (g/L)	35–52	Nu-femme™	44.43 ± 2.02	43.52 ± 2.54^**^
		Placebo	43.92 ± 2.27	43.71 ± 2.56
Aspartate aminotransferase (U/L)	<31	Nu-femme™	20.15 ± 5.73	19.26 ± 6.59^*^
		Placebo	21.41 ± 5.43	20.78 ± 5.52
Alanine aminotransferase (U/L)	<39	Nu-femme™	19.52 ± 7.37	19.12 ± 9.62
		Placebo	21.15 ± 10.36	21.36 ± 12.59
Gamma-glutamyl transferase (U/L)	<36	Nu-femme™	18.72 ± 17.10	18.52 ± 15.02
		Placebo	20.98 ± 12.34	19.40 ± 12.49
Systolic blood pressure (mm Hg)^a^	<130	Nu-femme™	114.93 ± 14.06	116.96 ± 17.32
		Placebo	119.32 ± 14.40	120.13 ± 15.67
Diastolic blood pressure (mm Hg)^a^	<85	Nu-femme™	73.93 ± 8.96	74.28 ± 10.74
		Placebo	75.93 ± 9.38	77.04 ± 11.04
Heart rate (BPM)^b^	60–100	Nu-femme™	71.02 ± 9.67	69.17 ± 7.52
		Placebo	71.95 ± 9.50	71.17 ± 8.76
Weight (kg)	–	Nu-femme™	74.70 ± 18.07	73.70 ± 16.85
		Placebo	75.91 ± 17.87	76.29 ± 19.50
BMI (kg/m^2^)	–	Nu-femme™	28.48 ± 6.58	28.01 ± 6.11
		Placebo	28.67 ± 6.16	28.90 ± 6.84

^*^P < 0.05, ^**^P < 0.01, significant within-group. Within-group *P*-values generated by the paired *t*-test or Wilcoxon Signed Rank test. Ties or zeros excluded from Wilcoxon tests.

CBC: complete blood count; CMP: comprehensive metabolic panel.

a Reference values obtained from (43).

b Reference values obtained from (44).

### Adverse events

In this clinical study, there were a total of 128 AEs reported by 66 participants. Of these, 63 AEs reported by 37 participants were in the Nu-femme™ group and 65 AEs reported by 29 participants were in the placebo group. None of the AEs were categorised as ‘likely related’ or ‘related’. No participants required medical treatment or hospitalisation, and all AEs were resolved by the end of the study. No severe AEs were noted in either of the groups, with the AEs reported including headache, flu-like symptoms and abdominal pain in both placebo and Nu-femme™ groups.

## Discussion

There were no significant differences in the primary outcome after Nu-femme™ supplementation when compared to the placebo, with both groups experiencing a significant decrease in hot flush symptoms. Similar results were observed previously in a pilot trial of 63 postmenopausal women supplemented with *L. pumila* or a placebo, with the lack of effect attributed to the small sample size (27). A subsequent clinical study that enrolled 102 participants in the *L. pumila* group and 95 in the placebo group (16) demonstrated that *L. pumila* supplementation improved cardiovascular risk factors, sleep patterns and depressed mood in pre- and postmenopausal women (16). Hot flush symptom scores and QoL being a subjective measure are associated with high SDs allowing for confounding study results.

It is pertinent to mention that the placebo effect was significant in this study, as improvements in several domains were realised in the placebo group as well. The placebo effect of 58% noted in the current study is in agreement with that observed in the literature, and when a menopause-specific QoL questionnaire was used in a demographically similar population (28). The placebo effect in menopause studies may range from 1 to 59% with phytoestrogens (29), which is similar to the placebo effect noted with HRT for vasomotor symptoms (30) and a clinically significant 33% placebo effect has been observed with pharmaceutical products (31). To mitigate the placebo effect on menopausal symptoms especially on hot flush a sub-group analysis based on hot flush severity (at screening) was done. The Nu-femme™ supplementation alleviated hot flush symptoms by a significant 15% over the placebo in a subset of individuals with a high flush score (>20) at baseline. Since the minimal clinically important difference in hot flushes is approximately 50% (32), Nu-femme™ supplementation achieved this effect in this subgroup of women as the percent improvement was >50% in both groups, but the difference between them was 15% favouring the Nu-femme™ group.

Dramatic changes in hormonal balance occur during peri-menopause, with the decrease in oestrogen and increase in FSH and LH hormones ultimately reducing the level of progesterone, eventually causing permanent amenorrhea or menopause (33). FSH, which generally increases with the onset of menopause, was significantly suppressed in the Nu-femme™ group at weeks 12 and 24, but not in the placebo group at any time point, which may indicate a positive effect on improvement in menopausal symptoms. LH also showed a similar pattern with significant reduction in the Nu-femme™ group, indicating improvements in the hormonal profile during menopause. This improvement was not observed in a previous study where pre- and postmenopausal women were supplemented with 400 mg of *L. pumila* for 16 weeks (16), possibly indicating the effect of *E. longifolia* in assisting hormonal balance in pre- and postmenopausal women. Based on the sub-group analysis, women with high levels of free testosterone at baseline, indicating a low risk of cardiovascular events, showed a significant 15% improvement in hot flush symptoms over the placebo after Nu-femme™ supplementation. Furthermore, the stable concentration of total testosterone in the Nu-femme™ group suggests that, in comparison to the placebo where total testosterone levels decreased, maintaining the levels in the Nu-femme™ group is advantageous as observed with Nu-femme™ supplementation in this study. The positive effect on maintaining the testosterone balance may be attributed to the actions of the constituents of *E. longifolia* since similar results were obtained in a previous study (34). Another study findings support this, where both total and free testosterone increased significantly in senior women when supplemented with 400 mg of *E. longifolia* for 5 weeks (35). In conclusion, the hormonal profile showed an overall improvement in the Nu-femme™ group compared to the placebo.

There were no significant differences between- and within-group of bone markers for osteoporosis reflecting bone formation (BSAP) and resorption (NTX) (36). However, there was a nearly 10-fold decrease in NTX in the Nu-femme™ group (reduced by 4.7%) compared to the placebo group (reduced by 0.5%), indicating that bone collagen was undergoing less degradation, therefore, more resorption with Nu-femme™ supplementation. The reduction in NTX level cannot be assumed to be clinically insignificant since a similar reduction was achieved in postmenopausal women in a 6-week smoking cessation programme (37). Both *E. longifolia* and *L. pumila*have demonstrated a protective effect on bone loss due to the osteoporosis in previously published studies (37–41). A pre-clinical study on rats supports the positive effects of *E. longifolia*on bone turnover in androgen-deficient rats, based on the study quassinoid-rich *E. longifolia* extract enables to protect against bone loss due to testosterone deficiency (38) The proandrogen properties of *E. longifolia* stimulated osteoblast proliferation and differentiation, resulting in increased bone formation rate. *E. longifolia* also contains high concentrations of superoxide dismutase, an antioxidant that plays an important role in reducing bone loss and maintaining bone formation rate (39). *L. pumila* supplementation in overiectomised rats was able to improve femoral strength (35), which was related to the anti-oxidative property of the herb (41). As *E. longifolia* and *L. pumila*herbal ingredients in Nu-femme™ could be beneficial in bone health, Nu-femme™ benefits on alleviating bone loss due to menopause should be further studied in future.

A decreasing trend in serum LDL, cholesterol and triglycerides was observed after 12 weeks of supplementation in Nu-femme™ group, whereas no changes were observed in placebo group. A significant decrease in serum triglycerides and LDL was observed at week 12 in Nu-femme™ group compared to baseline; however, the decrease was not significant at week 24 compared to baseline, which may reflect the high variation between participants in this study. The mean BMI for Nu-femme^TM^and placebo is 28.24 and 29.53 kg/m^2^, respectively, which is higher than healthy BMI 25 kg/m^2^ [42].The higher mean BMI suggests that most of the subjects enrolled in this study might be overweight, and a fixed dosage of two capsules of Nu-femme™ might not be adequate to produce significant and consistent improvement in lipid profile and bone markers. Clinically relevant changes in blood parameters and serious AEs were not observed in this study, with Nu-femme^TM^ found to be safe and well tolerated in this population.

This study had a high placebo effect, which is common in menopause research. It is possible that dietary confounders may have contributed to the high placebo effects; future studies should potential confounder. Future studies should examine the urinary levels of phytoestrogens. Further, exercise regimes may have had a salutary effect on the QoL of participants that should be accounted for. Smoking is associated with greater frequency of hot flashes; therefore, this is another potential confounder to be considered in future studies. Despite potential confounders and the placebo effect, a subset of participants were identified, who responded significantly to Nu-femme™ compared to the placebo. Since there are several variables that can affect results of a clinical study, well-designed randomised controlled trial, including women with menopausal symptoms of defined severity, of specific race and distinct hormonal profile, may provide a responder population that achieves the desired outcomes of the study, including one which has hot flush symptoms >20 as inclusion criteria.

## Conclusions

There were no significant differences in mitigating the severity of hot flush symptoms, as well as QoL, hormone and lipid profiles, bone health markers, sleep and vitality parameters in healthy peri- and postmenopausal women supplemented with Nu-femme™ compared to the placebo. Nonetheless, Nu-femme™ was found to be safe and well tolerated in this population tested in the 6-month study, with no evidence of clinically significant baseline changes in any haematology and clinical chemistry parameters.

Though this study had a high placebo effect, which is in common with most studies related to menopause, herbal formulation consists of *L. pumila* and *E. longifolia* (Nu-femme™) may support reduction in hot flushes, improvements in hormone and lipid profile in healthy peri-menopausal and menopausal women.

## Declarations

### Ethics Approval and Consent to Participate

Ethics (IRB) approval was obtained from IRB Services, Aurora, ON, Canada on 15 September 2014 prior to initiation of any study-related activities.
